# Shao Di Pa Ning Decoction Attenuates Neuroinflammation in Parkinson’s Disease via PI3K/AKT Signaling Pathway

**DOI:** 10.1155/mi/6450498

**Published:** 2026-08-30

**Authors:** Peng Huang, Zhengqi Tao, Jingjing Hu, Wei Dong, Meixia Wang, Han wang, Ting Dong, Yun Zhang, Xiao Liang, Ning He, Wenming Yang, Shuangying Gui, Yuzhe Huang

**Affiliations:** ^1^ Department of Neurology, The First Affiliated Hospital of Anhui University of Chinese Medicine, Hefei 230031, Anhui, China, ahtcm.edu.cn; ^2^ Department of Pharmacy, Anhui University of Chinese Medicine, Hefei 230012, Anhui, China, ahtcm.edu.cn; ^3^ Institute of Pharmaceutics, Anhui Academy of Chinese Medicine, Hefei 230012, Anhui, China, ahtcm.edu.cn; ^4^ Key Laboratory of Xin’an Medicine, Ministry of Education, Hefei 230012, Anhui, China, moe.edu.cn; ^5^ Center for Xin’an Medicine and Modernization of Traditional Chinese Medicine, Institute of Health and Medicine Hefei Comprehensive National Science Center, Hefei 230012, Anhui, China

**Keywords:** neuroinflammatory, Parkinson’s disease, PI3K/AKT signaling pathway, Shao Di Pa Ning Decoction, transcriptomic

## Abstract

**Background:**

Parkinson’s disease (PD) is a neurodegenerative disorder with limited therapeutic options. Shao Di Pa Ning Decoction (SDPND), a traditional Chinese medicine (TCM) compound formula for PD, has demonstrated therapeutic efficacy, but its underlying mechanisms remain unclear.

**Purpose:**

This research aims to systematically investigate the mechanisms of SDPND, focusing on its anti‐inflammatory effects and related pathways in PD.

**Methods:**

The bioactive components of SDPND were first characterized to establish its material basis. Subsequently, A53T mice were administered SDPND to evaluate therapeutic improvements in motor dysfunction and neuroinflammatory responses, followed by transcriptomic profiling to identify potential pathways. Network pharmacology analysis was employed to validate biological pathways associated with these therapeutic effects. Molecular docking was used to study binding interactions between components and inflammatory, thereby offering mechanistic insights, with western blotting ultimately confirming the modulation of PI3K/AKT pathway activity.

**Results:**

Chemical composition analysis revealed 91 compounds. SDPND alleviated motor deficits and mitigated neuroinflammatory responses in A53T mice. Transcriptomic profiling revealed significant downregulation of the PI3K/AKT pathway. Network pharmacology analyses independently reaffirmed the enrichment of PI3K/AKT signaling as a pivotal hub. Molecular docking demonstrated robust binding affinities between bioactive components and core targets within the PI3K/AKT pathway, while western blotting analysis confirmed SDPND mediated suppression of PI3K/AKT phosphorylation, further validating pathway inhibition as a key mechanistic driver.

**Conclusion:**

Our findings first revealed a novel anti‐neuroinflammatory mechanism of SDPND via PI3K/AKT pathway suppression, which underlies its efficacy in mitigating PD symptoms. This discovery provides a robust theoretical foundation and pinpoints a strategic target for refining clinical PD therapeutics.

## 1. Introduction

Parkinson’s disease (PD) is the second most prevalent neurodegenerative disorder globally, characterized by the gradual degeneration of dopaminergic neurons in the substantia nigra and a reduction in dopamine levels within the striatum. Clinically, PD presents with motor symptoms like resting tremor, bradykinesia, and muscle rigidity, as well as non‐motor symptoms, which include cognitive impairment, autonomic dysfunction, and a range of other neuropsychiatric manifestations [1, 2]. The aberrant aggregation of α‐synuclein (α‐Syn) constitutes a primary pathogenic driver in the molecular pathology of this disorder, and misfolded α‐Syn evolves into Lewy bodies through the formation of oligomers in β‐sheet conformation, which triggers an imbalance of protein homeostasis and synaptic dysfunction in neurons [3, 4]. However, due to the pathologic heterogeneity of PD and the heterogeneous clinical manifestations, the lack of early biomarkers and precise therapeutic targets are still a major challenge for clinical diagnosis and treatment. Existing pharmacological strategies, such as levodopa replacement therapy, dopamine agonists, and anticholinergic drugs, can improve motor symptoms in the short term, but cannot slow down the process of neurodegeneration, and long‐term use is prone to induce end‐dose phenomenon, anisotropy, and other complications. Therefore, it is crucial to develop innovative therapeutic approaches based on clinical needs to stop disease progression by targeting the key pathological aspects of PD.

The Huangdi Neijing and other ancient texts refer to PD as “tremor syndrome.” Accumulating clinical evidence and studies have highlighted the neuroprotective effects of traditional Chinese medicine (TCM) in mitigating disease progression PD, which is gradually being recognized by clinicians and patients. Therefore, many studies in recent years have revealed the mechanisms and targets of TCM in the treatment of PD. The therapeutic potential of TCM in PD is their polypharmacological profile, enabling simultaneous modulation of multiple pathological targets through diverse bioactive constituents [5]. For instance, by suppressing peripheral immune cytokine production and attenuating neuroinflammatory infiltration, Di Huang Yin has been demonstrated to confer dopaminergic neuroprotection and ameliorate motor deficits in PD [6]. Qiji granules exert neuroprotective effects by inhibiting dopaminergic neuron apoptosis, as validated in the chronic PD model [7]. Huanglian Tang attenuates paraquat‐induced mitosis in the cell experiment, highlighting its therapeutic potential [8].

Shao Di Pa Ning Decoction (SDPND) is an empirical formula developed under the guidance of the Xin’an Medical theory. Its development was guided by the principle of “consolidating the root and cultivating the vital essence.” The consolidating basis is an important principle of treatment and health maintenance in TCM. It emphasizes strengthening the body’s energy and preventing and treating diseases by tonifying the body’s fundamentals and vital energy, which is an important guide to the clinical practice of modern Chinese medicine. Evidence from clinical observational studies support SDPND’s efficacy in tonifying the liver and kidney and activating blood circulation to resolve stasis, modestly ameliorating motor dysfunction in PD patients [9]. SDPND is composed of six herbs: *Paeonia lactiflora* Pall. (BS), *Rehmannia glutinosa* Libosch. (SD), *Lycium barbarum* L. (GQ), *Cistanche deserticola* Y. C. Ma (RCR), *Pheretima aspergillum* (DL), and *Spatholobus suberectus* Dunn (JXT). Alongside the traditional therapeutic actions of yin nourishment and blood replenishment, BS and SD exhibit anti‐inflammatory and analgesic pharmacological properties [10]. GQ and RCR are used to nourish the kidney and moisten dryness, and their pharmacological activities include immunomodulation and neuroprotection [11]. DL and JXT were used to activate the meridians and collaterals [12].

The pathogenesis of PD involves complex interactions among multiple pathological mechanisms, including the vicious cycle of oxidative stress‐mitochondrial dysfunction, impaired autophagy‐lysosomal pathways, and neuroinflammatory cascades [13–15]. Recent advances in transcriptomic, single‐cell sequencing, and multiomics studies have further demonstrated that chronic neuroinflammation and disease‐associated microglial activation are key drivers of PD progression, interacting closely with mitochondrial dysfunction and defective autophagy to accelerate dopaminergic neuronal degeneration [16]. In comparison to other signaling pathways, the PI3K/AKT pathway is more intricate, with its components and biological functions playing a crucial role in a variety of cellular processes, including cell survival, proliferation, metabolism, and inflammation [17]. This signaling pathway has been extensively studied and shown to have significant effects on the central nervous system [18]. Accumulating evidence from recent studies suggests that PI3K/AKT signaling serves as an important molecular hub linking neuroinflammation, oxidative stress, mitochondrial homeostasis, and autophagic flux, making it an attractive therapeutic target for neurodegenerative diseases, including PD [19]. Previous studies have demonstrated that targeting the PI3K/AKT pathway may offer therapeutic strategies for neuroinflammation‐related disorders. For instance, *Eucommia ulmoides* leaf extract exerts neuroprotective effects against oxidative stress‐associated neurodegeneration by activating the PI3K/AKT/GSK‐3β/Nrf2 axis [20], and ginsenoside inhibits microglia‐mediated neuroinflammation through the PI3K/AKT/NF‐κB signaling cascade. Recent pharmacological studies have further confirmed that modulation of the PI3K/AKT pathway effectively suppresses microglial activation, attenuates inflammatory cytokine release, and protects dopaminergic neurons in experimental PD models, highlighting the translational potential of this pathway [21, 22]. Furthermore, activated microglia exacerbate neuroinflammation in the striatum and substantia nigra by releasing excessive amounts of pro‐inflammatory cytokines such as TNF‐α, IL‐6, and ROS [23, 24]. Emerging evidence also indicates that sustained activation of pro‐inflammatory microglia disrupts neuronal homeostasis and contributes to progressive neurodegeneration through persistent inflammatory signaling and oxidative injury [25]. However, the neuroprotective efficacy of SDPND in PD remains to be elucidated.

In this research, UPLC‐Q‐Orbitrap‐MS was first employed to characterize its chemical components. Subsequently, in vivo experiments were carried out to validate the therapeutic efficacy of SDPND in PD, while network pharmacology was employed to identify potential active ingredients and predict their therapeutic targets, further supporting the rationale for SDPND’s clinical application.

## 2. Materials and Methods

### 2.1. Materials and Reagents

All traditional herbal medicines listed in Table 1 were provided by the First Affiliated Hospital of Anhui University of Chinese Medicine; Levodopa and Benserazide Hydrochloride tablet were purchased from Shanghai Roche Pharmaceuticals Ltd. ELISA kits were purchased from Jianglai Biological (Shanghai, China).

**Table 1:** The information on purchased Chinese medicine.

No.	Latin name	English name	Chinese name	Batch No.
1	*Paeonia lactiflora* Pall.	Paeoniae Radix Alba	Bɑishɑo	2310050094
2	*Rehmannia glutinosa* Libosch.	Rehmanniae Radix Praeparata	Shudihuɑng	2311280182
3	*Lycium barbarum* L.	Lycii Fructus	Gouqi	2311060132
4	*Cistanche deserticola* Y. C. Ma	Cistanches Herba	Roucongrong	240116023
5	*Pheretima aspergillum* (E. Perrier)	Pheretima	Dilong	2312080912
6	*Spatholobus suberectus* Dunn	Spatholobi Caulis	Jixueteng	2310310532

### 2.2. Preparation of SDPND

Weighing 60 g Radix Paeoniae Alba, 30 g radix rehmanniae preparata, 40 g *Lycium barbarum*, 30 g *Cistanche* herbal, 24 g *Pheretima vulgaris*, and 30 g Caulis Spatholob. The sample was decocted with 400 mL water for 1 h, filter, and the filtrate was collected. Add another 400 mL of water and decoct for 1 h and collect the filtrate. Both filtrates were combined, concentrated to a solution with an original herb content of 2.14 g/mL, and preserve at 4°C.

### 2.3. Animals and Treatments

A53T mice were divided into five groups randomly (*n* = 6): the A53T mice group (Model), the Levodopa and Benserazide Hydrochloride group (LBH, 0.1 g/kg), the SDPND low‐dose group (SDPND‐L, 7.0 g/kg), the SDPND medium‐dose group (SDPND‐M, 14.0 g/kg), and the SDPND high‐dose group (SDPND‐H, 28.0 g/kg); another 6 age‐matched male C57BL/6 mice served as the sham group. The LBH and SDPND groups received the specified doses via orally, while the Model and Sham groups were administered saline for 28 consecutive days. The transgenic mice are B6; C3‐Tg (Prnp‐SNCA ^∗^A53T)83Vle/J (A53T; JAX:004479). All experiments were performed on male mice aged 10 months and with a body weight of 20–25 g, which were purchased from the Shanghai Model Organisms Center, Inc (Shanghai, China; license number: SCXK (Hu) 2017–0010). The age‐ and gender‐matched C57BL/6J mice were used as a control. Mice were group‐housed under SPF conditions with a 12‐h light/dark cycle at a controlled temperature of 22 ± 2°C and relative humidity of 50% ± 5%. The mice had free access to water and food. The Experimental Animal Ethics Committee of Anhui University of TCM approved all experiments (No. AHUCM‐mouse‐2022026).

### 2.4. Identification of Major Chemical Components of SDPND by UPLC‐Q‐Orbitrap‐MS

The SDPND extract was diluted four‐fold with water, vortexed for 1–2 min, and then centrifuged twice at 14,000 rpm for 10 min each. The supernatant was collected for analysis.

Component analysis was performed using a Thermo Fisher Vanquish UHPLC system. Separation was achieved on a Waters ACQUITY UPLC HSS T3 column (2.1 mm × 100 mm, 1.8 μm) with a mobile phase composed of 0.1% formic acid in water (A) and acetonitrile (B). The gradient elution program was as follows: 0–25 min, 5%–18% B; 25–30 min, 18%–26% B; 30–40 min, 26%–57% B; 40–45 min, 57%–95% B; and 45–50 min, 95% B. The injection volume was 2 μL, the flow rate was set to 0.20 mL/min, and the column temperature was maintained at 30°C.

Mass spectrometry analysis was performed using an ESI source configured for dual‐polarity detection (positive/negative ion modes). Instrument parameters were optimized as follows: ion source conditions (spray voltage: ±3.5 kV; capillary temperature: 350°C; source heater: 400°C), gas flow settings (sheath gas: 35 psi; auxiliary gas: 10 psi), and lens voltages (tube lens offset: −110 V). Data acquisition was carried out in the Full Scan/dd‐MS^2^ mode, with full MS scans performed at a resolution of 70,000 (scan range *m/z* 100–1000).

### 2.5. Behavior Tests

#### 2.5.1. Pole Test

To evaluate locomotor bradykinesia in mice, a pole‐climbing test was performed. A plastic rod (1 cm in diameter and 60 cm in height), wrapped with gauze to prevent slipping, was positioned vertically, with a plastic ball (1.5 cm in diameter) attached to its top. Prior to testing, mice were acclimatized by placing them on a ball for 5 min. Rod descent latency (time for mice to descend with their forepaws grounded) was recorded to quantify motor coordination deficits and bradykinesia.

#### 2.5.2. Open Field Test

The open field test was used to assess spontaneous locomotor and exploratory behaviors in mice, helping to evaluate anxiety‐like responses in novel environments. The setup consists of a 45 cm × 45 cm × 45 cm arena, a camera system, and video analysis software. Mice acclimatize to the experimental room for 30 min pre‐test. During the experiment, we ensured that the experimental environment was quiet, with appropriate and consistent light, temperature and humidity. Each mouse was placed in the center of the arena, and its behavior was recorded for 5 min. The movement time and distance in the central versus peripheral zones were then analyzed. The arena was sanitized with 75% ethanol between trials and allowed to dry completely before reuse.

### 2.6. Hematoxylin and Eosin (H&E) Staining

Brain tissues were fixed in 4% paraformaldehyde and then transferred through a graded ethanol series for dehydration, followed by xylene for clearing. When the tissues became transparent, they were immersed in molten paraffin wax for 2 h to ensure complete infiltration. After solidification, the samples were sectioned, stained, and examined under a light microscope. A blinded histopathological scoring system was adopted to objectively evaluate the degree of tissue damage. Specifically, H&E‐stained brain tissue sections were independently evaluated by two investigators who were blinded to the experimental group assignments using predefined histopathological criteria. The histological scores were then subjected to statistical analysis. According to the predefined semi‐quantitative scoring criteria, pathological changes, including neuronal morphology, inflammatory cell infiltration, tissue structural integrity, and vacuolar degeneration, were comprehensively evaluated. Histopathological damage was graded using a 4‐point scale: grade 0, normal tissue structure; grade 1, mild pathological changes; grade 2, moderate pathological changes; and grade 3, severe pathological changes. The final histopathological score for each sample was calculated as the average of the independent scores assigned by the two investigators and was used for subsequent statistical analysis.

### 2.7. Measurement of TNF‐α and IL‐6 Levels

According to the manufacturer’s instructions, TNF‐α and IL‐6 levels in the serum were determined using ELISA kits.

### 2.8. Immunofluorescence

Immunofluorescence was conducted to examine the expression of Iba1 and CD68 in the substantia nigra and striatum. Brain tissue sections were permeabilized with 0.3% Triton X‐100 in PBS for 30 min at room temperature (RT), followed by blocking with 5% goat serum in PBS for 1 h at RT. Sections were then incubated overnight at 4°C with primary antibodies: Iba1 (WAKO, #019‐19741, 1:500) and CD68 (Abcam, #ab125212, 1:100). Following washing, sections were incubated with Alexa Fluor 488‐conjugated goat anti‐rabbit IgG (ZEN‐BIOSCIENCE, #550,043, 1:500) for 2 h at RT and then imaged by confocal microscopy. Quantitative analysis of immunofluorescence images was performed using ImageJ software. Iba1 served as a microglial marker, and CD68 identified the M1‐activated phenotype. The expression level of M1 microglia was quantified by calculating the percentage of CD68^+^Iba1^+^ double‐positive cells among the total Iba1^+^ cells.

### 2.9. Transcriptomics

Total RNA was extracted from the brain tissues (*n* = 3). RNA integrity was verified using 1% agarose gel electrophoresis and a Bioanalyzer (RIN ≥ 6.5), with purity assessed by measuring the absorbance ratios (OD260/280: 1.8–2.2 and OD260/230 ≥ 2.0). Poly(A)+ mRNA was enriched and fragmented. Double‐stranded cDNA libraries were prepared using TruSeq kits, followed by library quantification and size‐selection (300–400 bp). Sequencing was performed on NovaSeq platforms.

### 2.10. Screening of the Main Targets of Action of SDPND

Based on the results of UPLC‐Q‐Orbitrap‐MS, the main active ingredients of SDPND were identified by querying the TCM Systems Pharmacology (TCMSP) database (https://old.tcmsp-e.com/tcmsp.php). Therapeutic targets of the ingredients were predicted via the Swiss Target Prediction platform (http://www.swisstargetprediction.ch/). Finally, the UniProt database was employed to screen and validate the primary targets of the SDPND components.

### 2.11. Protein–Protein Interaction (PPI) Network Construction

Targets related to PD were retrieved from online databases, such as OMIM (https://omim.org/) and GeneCards (https://www.genecards.org/). After removing duplicate genes, we intersected SDPND’s primary targets with PD‐related genes using Venny 2.1.0 and visualized the overlap in a Venn diagram. Also, these targets were constructed a PPI network.

### 2.12. Molecular Docking

From the TCMSP and PubChem databases, 3D structural data of bioactive compounds were acquired and archived in the mol2 format. Corresponding protein structures were acquired from the Protein Data Bank. Molecular docking was conducted using CB‐Dock 2 (https://cadd.labshare.cn/cb-dock2) with three independent replicates per docking group, followed by structural visualization in PyMOL 2.3. Optimal binding conformations were determined by analyzing docking energy scores, where lower binding free energy values were associated with enhanced stability of ligand–receptor complexes.

### 2.13. Western Blotting

Substantia nigra and striatum tissues (~0.1 g each) were homogenized in RIPA buffer (600 µL) and centrifuged (12000 g, 15 min) to collect supernatants. Lysates were denatured by mixing with 5× SDS buffer at a 4:1 ratio, heated at 100°C for 15 min, electrophoresed (80 V, 1 h), and transferred to PVDF membranes using a wet transfer system. After blocking with 5% skim milk in TBST for 2 h at RT, membranes were incubated overnight at 4°C with primary antibodies: PI3K (Abcam, ab86714, 1:1000), p‐PI3K (Abcam, ab18265, 11:1000), AKT (CST, 4691 s, 1:1000), p‐AKT (CST, 4060 s,1:2000), and β‐actin (Zsbio, TA‐09, 1:2000). HRP‐conjugated secondary antibodies (1:20,000) were applied for 1.5 h at RT. Protein bands were visualized using an ECL substrate (Shandong Sparkjade Biotechnology Co., Ltd.) and a chemiluminescence detection system, with quantification performed by ImageJ.

### 2.14. Statistical Analysis

Statistical analyses were conducted using GraphPad Prism 9.5. Quantitative data are expressed as the mean ± standard deviation (SD). Intergroup comparisons were assessed as follows: Student’s *t*‐test for evaluating statistical significance between two experimental groups and one‐way analysis of variance (ANOVA) for multigroup comparisons.

## 3. Results

### 3.1. SDPND Chemical Components Analysis

**Figure 1:**
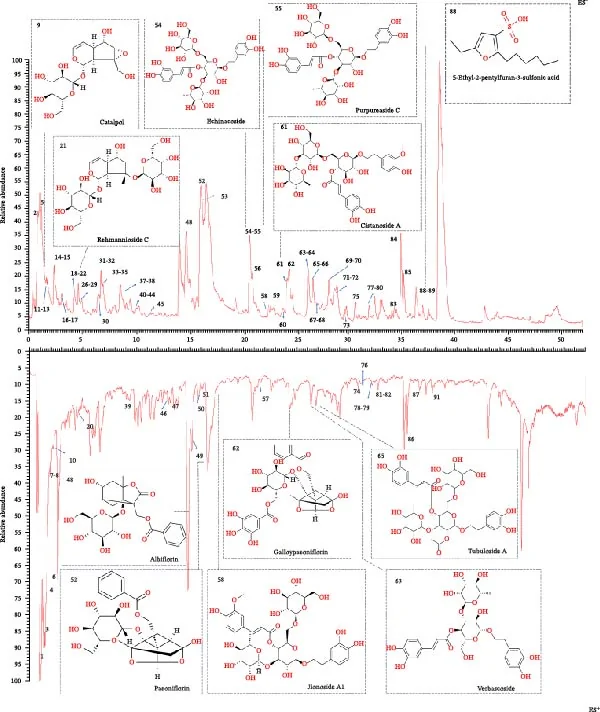
Total ion current of SDPND in negative and positive mode.

In this experiment, 91 components in SDPND were identified using UPLC‐Q‐Orbitrap‐MS and analyzed with Xcalibur. The mass spectra in both positive and negative ionization modes, along with major constituents such as albiflorin, paeoniflorin, and cistanoside A, are presented in detail (Figure 1). The related information of each chemical component is shown in Table 2.

**Table 2:** Compound compositions in SDPND.

No	RT (min)	Formula	Type of compound	Identification	Adductive type	Calculated (*m*/*z*)	Detected (*m*/*z*)	Error (ppm)	MS/MS	Source
1	0.70	C_6_H_14_N_2_O_2_	Amino acid	Lysine	[M + H]+	147.1128	147.1129	0.68	130.0863, 84.0807	DL
2	0.95	C_12_H_18_O_11_	Organic acids	Ascorbic acid 2‐glucoside	[M − H]−	337.07763	337.0778	0.50	277.0567, 157.0143, 113.9960	GQ
3	1.03	C_5_H_11_NO_2_	Alkaloid	Betaine	[M + H]+	118.08625	118.0862	−0.42	72.0808, 59.0730	GQ
4	1.17	C_6_H_5_NO_2_	Organic acids	Nicotinic acid	[M + H]+	124.0393	124.0394	0.81	96.0444, 82.0652, 80.0495, 55.0179	DL
5	1.31	C_6_H_8_O_7_	Organic acids	Citric acid	[M − H]−	191.01972	191.0197	−0.10	173.0092, 129.0192, 111.0088, 87.0088	SD
6	1.35	C_10_H_13_N_5_O_4_	Nucleotides	Adenosine	[M + H]+	268.10403	268.103	−3.84	136.0619	DL
7	1.50	C_9_H_11_NO_3_	Amino acid	p‐Hydroxyphenylalanine	[M + H]+	182.08116	182.0808	−1.98	165.0546,136.0757,123.0440	DL
8	1.56	C_5_H_4_N_4_O	Nucleotides	6‐Hydroxypurine	[M + H]+	137.04578	137.0459	0.88	119.0354,110.0349	DL
9	1.66	C_13_H_16_O_10_	Monoterpene glycosides	6‐O‐galloylglucose	[M − H]−	331.06706	331.0671	0.12	271.0483, 169.0143, 125.0245, 211.0241	BS
10	1.71	C_5_H_5_N_5_O	Nucleotides	Guanine	[M + H]+	152.05668	152.0568	0.79	135.0302,110.0350	DL
11	1.80	C_10_H_12_N_4_O_5_	Nucleotides	Inosine	[M − H]−	267.07349	267.0733	−0.71	135.0312,108.0203,92.0254	DL
12	1.91	C_15_H_24_O_10_	Iridoids	Dihydrocatalpol	[M − H]−	363.12967	363.1296	−0.19	96.9701, 169, 109.0295, 153.0557	SD
13	2.09	C_16_H_24_O_10_	Monoterpene glycosides	8‐debenzoylpaeoniflorin	[M + HCOO]−	421.1351	421.1349	−0.47	375.1296, 345.1187, 195.0663, 165.0557	BS
14	2.39	C_7_H_6_O_5_	Organic acids	Gallic acid	[M − H]−	169.01424	169.0141	−0.83	125.0244	BS
15	2.83	C_16_H_24_O_9_	Monoterpene glycosides	1‐O‐β‐D‐glucopyransoyl‐paeonisuffron	[M − H]−	359.13475	359.1343	−1.25	179.0713, 197.0826, 122.0372, 85.0295	BS
16	3.36	C_27_H_42_O_20_	Phenylethanoid Glycosides	Rehmannia D	[M − H]−	685.21966	685.2195	−0.23	505.1574, 179.0560, 221.0556, 263.0770	SD
17	3.39	C_21_H_32_O_15_	Iridoids	Melittoside	[M − H]−	523.16684	523.167	0.31	179.0561	SD
18	4.30	C_24_H_30_O_15_S	Sulfates	Mudanpioside E sulfite/isomer	[M − H]−	589.1232	589.1228	−0.68	421.0814, 259.0285, 167.0347	BS
19	4.41	C_20_H_30_O_12_	Phenylethanoid glycosides	Verbasoside/isomer	[M − H]−	461.16644	461.1672	1.65	315.1089, 113.0244, 135.0452, 161.0456, 179.0554	RCR
20	4.50	C_15_H_14_O_7_	Phenolics	Gallocatechin	[M + H]+	307.08122	307.0826	4.49	163.0391,139.0392	JXT
21	4.50	C_21_H_34_O_14_	Iridoids	Rehmannioside C	[M − H]−	509.18757	509.1883	1.43	179.0562,167.0349	SD
22	4.74	C_14_H_20_O_7_	Phenylethanoid Glycosides	Salidroside/isomer	[M − H]−	299.11362	299.1151	4.95	179.0561, 89.0244, 59.0138, 119.0506	RCR
23	5.13	C_16_H_24_O_10_	Iridoids	8‐Epiloganic acid/isomer	[M − H]−	375.12967	375.1295	−0.45	213.0768,169.0870,151.0764,59.0138	SD
24	5.13	C_16_H_24_O_10_	Iridoids	Adoxosidic acid/isomer	[M − H]−	375.12967	375.1295	−0.45	213.0769,169.0870,151.0764,59.0138	RCR
25	5.16	C_7_H_6_O_4_	Phenolics	Protocatechuic acid	[M − H]−	153.01933	153.0193	−0.20	109.0295	JXT
26	5.74	C_11_H_12_N_2_O_2_	Amino acid	Tryptophan	[M + H]+	205.09715	205.0975	1.71	188.0708,146.0602,118.0652	DL
27	6.17	C_15_H_24_O_9_	Iridoids	Ajugol	[M − H]−	347.13475	347.1341	−1.87	167.0714,123.0816	SD
28	6.26	C_16_H_24_O_10_	Iridoids	Adoxosidic acid/isomer	[M − H]−	375.12967	375.1313	4.35	167.0713, 149.0608	RCR
29	6.40	C_16_H_18_O_9_	Organic acids	Neochlorogenic acid	[M − H]−	353.0878	353.0877	−0.28	135.0452, 179.0351,191.0562	GQ
30	6.68	C_23_H_28_O_13_S	Sulfates	Paeoniflorin sulfite/isomer	[M − H]−	543.11778	543.1173	−0.88	497.1130, 421.0800, 375.0764	BS
31	6.72	C_20_H_30_O_12_	Phenylethanoid glycosides	Verbasoside/isomer	[M − H]−	461.16644	461.1688	5.12	116.9285, 315.1085, 135.0451	RCR
32	6.72	C_24_H_30_O_15_S	Sulfates	Mudanpioside E sulfite/isomer	[M − H]−	589.1232	589.1233	0.17	421.0814, 259.0287, 167.0350	BS
33	6.97	C_16_H_24_O_10_	Iridoids	8‐Epiloganic acid/isomer	[M − H]−	375.12967	375.1292	−1.25	213.0768,169.0870,151.0764,59.0138	SD
34	7.19	C_14_H_20_O_7_	Phenylethanoid glycosides	Salidroside/isomer	[M − H]−	299.11362	299.1127	−3.08	179.0568, 119.0508, 59.0245, 59.0139	RCR
35	7.21	C_16_H_24_O_10_	Iridoids	Adoxosidic acid/isomer	[M − H]−	375.12967	375.1297	0.08	213.0770,169.0872,151.0766	RCR
36	8.57	C_15_H_14_O_6_	Phenolics	(−)‐Catechin	[M − H]−	289.07176	289.0718	0.14	245.0820,203.0715,125.0244,109.0295	JXT
37	8.72	C_23_H_28_O_13_S	Sulfates	Paeoniflorin sulfite/isomer	[M − H]−	543.11778	543.1177	−0.15	497.1265, 421.0812, 375.0736	BS
38	8.82	C_16_H_18_O_9_	Organic acids	Chlorogenic acid	[M − H]−	353.0878	353.0881	0.85	191.0562, 179.0348	GQ
39	8.95	C_15_H_14_O_7_	Phenolics	(−)‐Epigallocatechin	[M + H]+	307.08122	307.0817	1.56	163.0392, 139.0392	JXT
40	10.03	C_21_H_32_O_12_	Phenylethanoid glycosides	Cistanoside E/isomer	[M − H]−	475.18209	475.1823	0.44	179.0557,113.0245	RCR
41	10.17	C_15_H_14_O_6_	Phenolics	Epicatechin	[M − H]−	289.07176	289.0719	0.48	245.0820,203.0714,125.0245,109.0296	JXT
42	10.23	C_16_H_18_O_9_	Organic acids	Cryptochlorogenic acid	[M − H]−	353.0878	353.0883	1.42	191.0561, 179.0355	GQ
43	10.43	C_23_H_28_O_12_	Monoterpene glycosides	Oxypaeoniflora	[M − H]−	495.15079	495.1508	0.02	137.0245,93.0346	BS
44	11.08	C_21_H_32_O_12_	Phenylethanoid glycosides	Cistanoside E/isomer	[M − H]−	475.18209	475.182	−0.19	113.0245,179.0564	RCR
45	11.47	C_9_H_8_O_4_	Organic acids	Caffeic acid	[M − H]−	179.03498	179.0352	1.23	135.0453	RCR
46	13.15	C_25_H_35_O_6_N_3_	Alkaloid	Scotanamine D	[M + H]+	474.25986	474.2604	1.14	457.2336,222.1129,165.0549,123.0442	GQ
47	13.23	C_25_H_33_O_6_N_3_	Alkaloid	N1‐caffeoyl‐N3‐dihydrocaffeoyl‐spermidinedi‐hex	[M + H]+	472.24421	472.2449	1.46	310.2131,220.0971,163.0392	GQ
48	14.98	C_23_H_28_O_11_	Monoterpene glycosides	Albiflorin	[M + HCOO]−	525.16136	525.1626	2.36	479.1546, 151.0767,121.0296	BS
49	15.18	C_25_H_33_O_6_N_3_	Alkaloid	N1‐dihydrocaffeoyl‐N3‐caffeoyl‐spermidinehex/isomer	[M + H]+	472.24421	472.245	1.67	310.2126,220.0976,163.0393	GQ
50	15.46	C_17_H_20_N_4_O_6_	Amino acid	Riboflavin	[M + H]+	377.14556	377.1462	1.70	243.0881,172.0871,216.0771	DL
51	16.14	C_25_H_33_O_6_N_3_	Alkaloid	N1‐dihydrocaffeoyl‐N3‐caffeoyl‐spermidine‐hex/isomer	[M + H]+	472.24421	472.2451	1.88	310.2130,222.1129,163.0393	GQ
52	16.29	C_23_H_28_O_11_	Monoterpene glycosides	Paeoniflorin	[M + HCOO]−	525.16136	525.1615	0.27	479.1531,327.1091,165.0562,121.0296	BS
53	16.46	C_35_H_46_O_21_	Phenylethanoid glycosides	Cistantubuloside C1/C2	[M − H]−	801.24588	801.2464	0.65	783.2360,639.2139,179.0352,161.0245	RCR
54	20.85	C_35_H_46_O_20_	Phenylethanoid Glycosides	Echinacoside	[M − H]−	785.25096	785.2515	0.69	623.2195,477.1632,161.0245	RCR, SD
55	20.92	C_35_H_46_O_20_	Phenylethanoid glycosides	Purpureaside C	[M − H]−	785.25096	785.2513	0.43	623.2190,477.1593,161.0244	DS
56	21.12	C_15_H_12_O_6_	Phenolics	Aromadendrin	[M − H]−	287.05611	287.0557	−1.43	151.0037,135.0452	JXT
57	21.32	C_15_H_12_O_6_	Phenolics	Eriodictyol	[M + H]+	289.07066	289.0712	1.87	179.0338,163.0392,153.0185,135.0443	JXT
58	22.71	C_36_H_48_O_20_	Phenylethanoid glycosides	Jionoside A1/A2	[M − H]−	799.26661	799.2664	−0.26	623.2196,477.1614,175.0401,160.0166	SD
59	23.10	C_16_H_26_O_8_	Phenylethanoid glycosides	Rehmapicroside	[M − H]−	345.1554	345.1552	−0.58	183.1020, 179., 165.0920	SD
60	24.01	C_27_H_30_O_16_	Flavonoids	Rutin	[M − H]−	609.1461	609.1458	−0.49	300.0274,271.0247,255.0298,151.0039	GQ
61	24.33	C_36_H_48_O_20_	Phenylethanoid glycosides	Cistanoside A	[M − H]−	799.26661	799.267	0.49	637.2343,491.1736,161.0244,	RCR
62	24.58	C_30_H_32_O_15_	Monoterpene glycosides	Galloylpaeoniflorin	[M − H]−	631.16684	631.1669	0.10	465.1397,313.0567,271.0457,169.0143	BS
63	26.33	C_29_H_36_O_15_	Phenylethanoid glycosides	Verbascoside	[M − H]−	623.19814	623.1982	0.10	461.1658,161.0244,133.0295	SD
64	26.42	C_29_H_36_O_15_	Phenylethanoid glycosides	Verbascoside	[M − H]−	623.19814	623.1981	−0.06	461.1658,315.1079,161.0244	RCR
65	26.65	C_37_H_48_O_21_	Phenylethanoid glycosides	Tubuloside A	[M − H]−	827.26153	827.2618	0.33	665.2303,623.2172,477.1581,161.0244	RCR
66	26.82	C_8_H_16_O_4_S	Sulfates	3,5‐Dimethylcyclohexyl hydrogen sulfate/isomer (5‐octenyl sulfate)	[M − H]−	207.06965	207.0696	−0.24	143.1075,129.0924,113.0970,79.9574	DL
67	27.30	C_8_H_16_O_4_S	Sulfates	3,5‐Dimethylcyclohexyl hydrogen sulfate/isomer (5‐octenyl sulfate)	[M − H]−	207.06965	207.0696	−0.24	143.1078,129.0921,113.0975,79.9574	DL
68	27.50	C_37_H_50_O_20_	Phenylethanoid glycosides	Jioglutoside B	[M − H]−	813.28226	813.2823	0.05	637.2338,619.2239,193.0505,175.0401,160.0166	SD
69	28.25	C_29_H_36_O_15_	Phenylethanoid glycosides	Isoacteoside	[M − H]−	623.19814	623.1985	0.58	461.1665,161.0245,135.0452	SD
70	28.35	C_29_H_36_O_15_	Phenylethanoid glycosides	Isoacteoside	[M − H]−	623.19814	623.1985	0.58	461.1665,315.1093,161.0245	RCR
71	28.83	C_24_H_30_O_12_	Monoterpene glycosides	Mudanpioside D	[M − H]−	509.16644	509.1664	−0.08	121.0295	BS
72	29.02	C_23_H_28_O_11_	Monoterpene glycosides	Mudanpioside I	[M − H]−	479.15588	479.1546	−2.67	121.0295	BS
73	29.64	C_30_H_38_O_15_	Phenylethanoid glycosides	Cistanoside C	[M − H]−	637.21379	637.214	0.33	475.1805, 161.0244	RCR
74	30.58	C_27_H_30_O_13_	Flavonoids	Glycyroside	[M + H]+	563.17591	563.1791	5.66	269.0816	JXT
75	30.63	C_31_H_38_O_16_	Phenylethanoid glycosides	2^′^‐acetylacteoside	[M − H]−	665.2087	665.2087	0.00	623.1965,503.1774,461.1661,315.1093,161.0244	RCR
76	30.97	C_22_H_22_O_9_	Flavonoids	Ononin	[M + H]+	431.13365	431.1346	2.20	269.0816	JXT
77	32.01	C_30_H_32_O_13_	Monoterpene glycosides	Benzoyloxypaeoniflorin	[M − H]−	599.17701	599.1777	1.15	137.0245, 121.0296, 93.0347	BS
78	32.05	C_15_H_10_O_4_	Phenolics	Daidzein	[M + H]+	255.06518	255.0659	2.82	227.0708,199.0759,137.0236	JXT
79	32.15	C_15_H_12_O_4_	Phenolics	Liquiritigenin	[M + H]+	257.08083	257.0815	2.61	147.0445, 137.0236	JXT
80	32.19	C_31_H_38_O_16_	Phenylethanoid glycosides	Tubuloside B	[M − H]−	665.2087	665.209	0.45	623.2000,461.1666,315.1092,161.0244	RCR
81	32.62	C_23_H_24_O_10_	Flavonoids	8‐methylretusin‐7β‐glucoside	[M + H]+	461.14422	461.146	3.86	299.0926, 284.0689	JXT
82	32.78	C_16_H_14_O_7_	Phenolics	6‐methoxyeriodictyol	[M + H]+	317.06667	317.0669	0.73	302.0434,181.0143,135.0453,165.9909	JXT
83	34.24	C_12_H_18_O_5_S	Sulfates	5‐Hexyl‐2,4‐dihydroxybenzenesulfonic acid	[M − H]−	273.08021	273.0803	0.33	193.1236,79.9575	DL
84	35.02	C_31_H_34_O_14_	Monoterpene glycosides	Mudanpioside B	[M − H]−	629.18757	629.188	0.68	121.0296	BS
85	35.13	C_30_H_32_O_12_	Monoterpene glycosides	Benzoylpaeoniflorin/isomer	[M − H]−	583.18209	583.1865	7.56	165.0559, 121.0296	BS
86	35.37	C_15_H_10_O_5_	Flavonoids	7,3^′^,4^′^‐trihydroxyflavone	[M + H]+	271.06009	271.0611	3.73	243.0663, 215.0710, 153.0188	JXT
87	36.49	C_15_H_12_O_4_	Phenolics	Isoliquiritigenin	[M + H]+	257.08083	257.0817	3.38	147.0446, 137.0238	JXT
88	36.78	C_11_H_18_O_4_S	Sulfates	5‐Ethyl‐2‐pentylfuran‐3‐sulfonic acid	[M − H]−	245.0853	245.0854	0.41	230.0627,188.0151,172.9914,79.9575	DL
89	36.96	C_30_H_32_O_12_	Monoterpene glycosides	Benzoylpaeoniflorin/isomer	[M − H]−	583.18209	583.1826	0.87	121.0296	BS
90	37.28	C_16_H_12_O_4_	Phenolics	Formononetin	[M − H]−	267.06628	267.06628	0.00	252.0430,223.0404	JXT
91	38.65	C_12_H_20_O_4_S	Sulfates	5‐Ethyl‐2‐hexylfuran‐3‐sulfonic acid	[M + H]+	259.10095	259.101	0.19	188.0150,172.9915,79.9575	DL

### 3.2. SDPND Improves Motor Function and Reduces Neuroinflammatory Infiltration in A53T Mice

Detailed drug administration design and experimental procedures are provided in the schematic diagram (Figure 2A). To investigate the effects of SDPND in mice, body weights were recorded across all groups. Results showed no significant differences compared to the sham group (Figure 2B).

Figure 2SDPND improves behavioral deficits in A53T mice. (A) Schematic diagram of experimental design. (B) Difference in body weight after drug administration. (C–G) Behavioral assessment. (C) Climbing time of each group of mice in the pole test. (D) Percentage of time spent in the center zone in open field test. (E) Plot of trajectories of each group of mice in the open field experiment. (F) Total distance traveled by each group of mice in open field test. (G) Percentage of distance covered by the central zone in open field test.  ^∗^ *p* < 0.05,  ^∗∗^ *p* < 0.01, vs. Sham group; ^#^ *p* < 0.05, ^##^ *p* < 0.01, vs. Model group. (H) Representative images of H&E staining.

**Figure:**
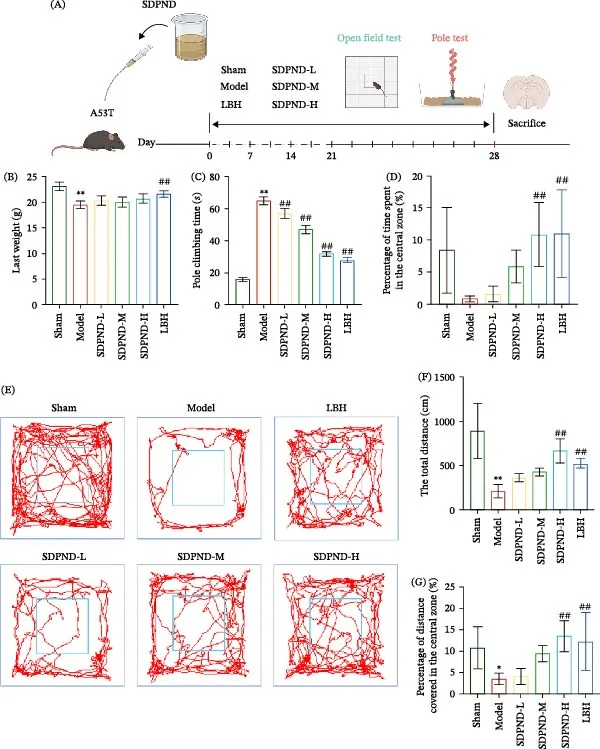

**Figure:**
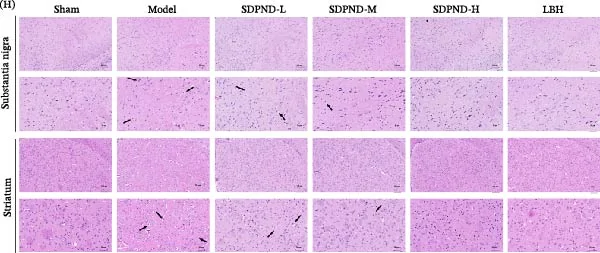

In the pole test, A53T mice demonstrated significantly impaired motor coordination compared to the Sham group, as indicated by a prolonged task completion time (*p* < 0.01). SDPND treatment effectively ameliorated motor coordination deficits across dose groups. The results showed that the climbing time of mice treated with SDPND was lower than that of the A53T group (*p* < 0.01), with the SDPND‐H group showing results comparable to the positive control group (Figure 2C), both significantly reducing movement time.

In the open field test, A53T mice displayed significant motor and exploratory deficits compared to the sham group, with a marked reduction in total distance traveled (*p* < 0.01) and a significant decrease in the ratio of center distance to total distance traveled (*p* < 0.05) during testing (Figure 2D,F). The LBH group partially reversed these deficits, showing an increase in total movement distance (*p* < 0.01) and a significant increase in central zone duration (*p* < 0.01). SDPND improved motor dysfunction in a dose‐dependent manner, with the high‐dose group demonstrating the most significant improvement (*p* < 0.01). Notably, motor function was nearly restored in the SDPND‐H group compared to the Model group (*p* < 0.01), and central zone movement duration returned to control levels (Figure 2G). Comparative locomotion trajectories of mice in the open field test are shown in Figure 2E.

H&E staining results showed that the overall structures of the substantia nigra and striatum in the sham group were basically normal, the neurons of tissues were neatly arranged, the structure were full, there was no obvious loose edema, and there was no inflammatory cell infiltration in the tissues. In the model group, the overall structures of the substantia nigra and striatum were irregular, the neurons of the tissues were arranged in a disordered manner, many neurons were sparsely edematous, and there were a large number of inflammatory cells infiltration. The damage was improved to varying degrees after the treatment of SDPND, and the brain tissues of the low‐dose group were abnormal, the neurons of the tissues were arranged in a disordered manner, and some of them were edematous, and the inflammatory cell infiltration could be seen. In the SDPND‐M group, the overall structure of the brain tissue was mildly abnormal, with disturbed arrangement of neurons, and a few neurons were sparse and edematous. In the SDPND‐H group, the structure of the brain tissue was normal, the neurons of the tissue were arranged in an orderly manner, there was no obvious edema, and very few inflammatory cells were seen (Figure 2H). The results of the blinded histopathological scoring are presented in Supporting Information, as shown in Figure S1. The above results indicated that SDPND could reduce PD‐induced brain tissue injury in mice.

### 3.3. SDPND Inhibits the Expression of M1‐Type Microglia

Microglia activation is a hallmark of neuroinflammation, characterized by polarization toward either the pro‐inflammatory M1 or anti‐inflammatory M2 phenotypes [26]. To assess the effect of SDPND on neuroinflammation, we performed immunofluorescence staining in the tissues of A53T mice using CD68 to label M1‐polarized microglia (Figure 3A,B). Quantitative analysis showed that CD68 expression was significantly reduced in both the SDPND and positive control groups compared to Model group (*p* < 0.05). Concurrently, we observed that microglial activation varied in a dose‐dependent manner following SDPND administration, with a notable reduction in the expression of the pro‐inflammatory phenotype, particularly in the SDPND‐M and SDPND‐H groups (*p* < 0.01). These data suggest that SDPND attenuates activated reactive glial cells in A53T mice (Figure 3C,D).

Figure 3Neuroprotective effect of SDPND in A53T mice. (A, B) Representative confocal microscopy images showing Iba1 (green) and CD68 (red) expression, with scale bars = 50 μm. (C) Quantitative co‐localization analysis demonstrated Iba1/CD68 microglial activation in the substantia nigra. (D) Statistical assessment revealed significant Iba1/CD68 co‐localization in the striatum region. (E) The levels of serum inflammatory factors TNF‐α and IL‐6 were detected by enzyme‐linked immunosorbent assay in each group of mice. (F) The levels of serum inflammatory factors IL‐6  ^∗^ *p* < 0.05,  ^∗∗^ *p* < 0.01, vs. Sham group; ^#^ *p* < 0.05, ^##^ *p* < 0.01, vs. Model group.

**Figure:**
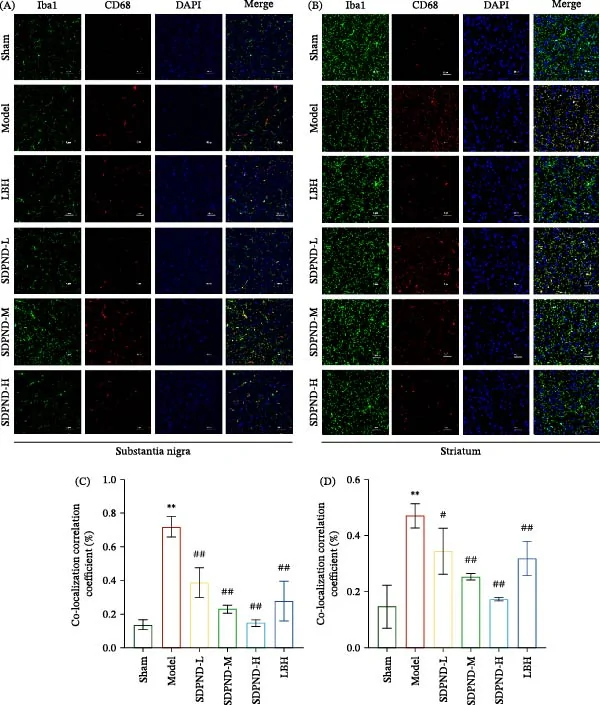

**Figure:**
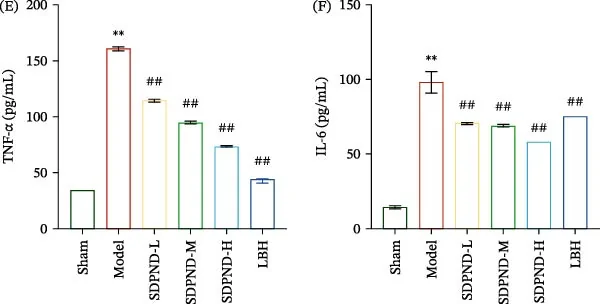

Activated pro‐inflammatory microglia release inflammatory cytokines, which may subsequently induce neuronal apoptosis. Serum analysis of A53T mice revealed elevated levels of both TNF‐α and IL‐6. Compared with the Model group, both SDPND and the LBH groups reduced the levels of inflammatory cytokines. This effect was particularly evident in TNF‐α measurements, where the LBH and SDPND‐H groups showed a marked downward trend, suggesting its potential to mitigate neuroinflammation (Figure 3E,F).

### 3.4. Effect of SDPND on Transcription in Brain Tissues of A53T Mice

The Venn diagram demonstrates that SDPND treatment reversed 41 differentially expressed genes (DEGs), which may represent potential therapeutic targets for PD (Figure 4A). Principal component analysis (PCA) revealed intra‐group clustering among the Sham, Model, and SDPND groups. Notably, a partial spatial overlap was observed between the Sham and SDPND groups (Figure 4B). Subsequently, the volcano plot depicted the global distribution of DEGs, showing significant expression differences between the Sham and Model groups, with 344 genes upregulated and 98 genes downregulated in the model group. Compared to the Model group, SDPND treatment significantly altered the expression of 210 genes, including 72 upregulated and 138 downregulated genes (Figure 4C). Meanwhile, the heatmap clearly illustrates the significant DEGs (Figure 4D).

Figure 4Effect of SDPND on transcription in A53T mice. (A) Diagrams of Venn displayed the regulation of DEGs by SDPND treatment principal component analysis of different groups. (B) Principal component analysis of different groups. (C) The DEGs were represented as a volcano map of model vs control and SDPND vs model. (D) Clustered heatmap for DEGs from different groups with rows representing DEGs and columns representing samples. Red color indicates that the gene is highly expressed in the sample, green color indicates that the gene is lowly expressed in the sample. (E) KEGG enrichment analysis of DEGs. (F) GSEA enrichment analysis of genes related to the PI3K/AKT pathway. (G) The heatmaps show the top 20 DEG expressions in the PI3K/AKT pathway.

**Figure:**
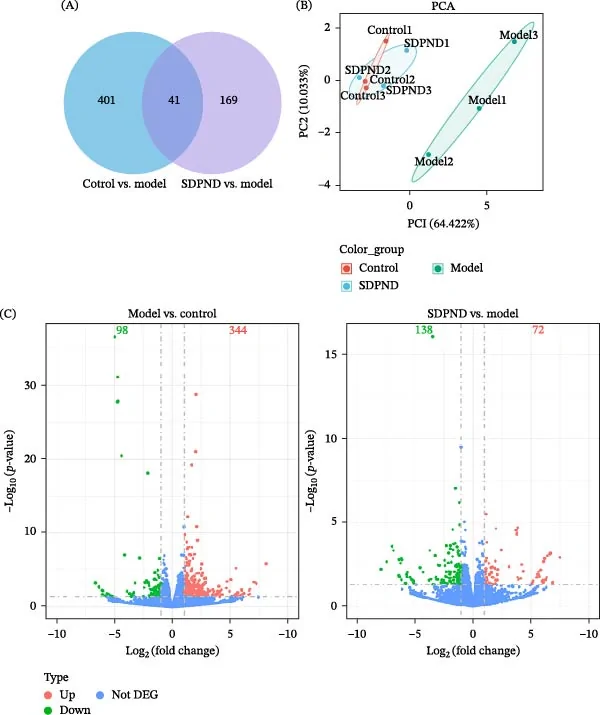

**Figure:**
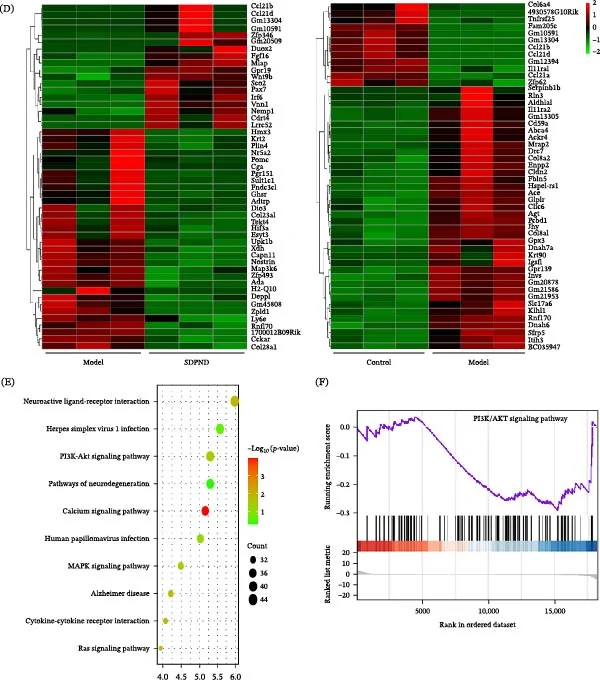

**Figure:**
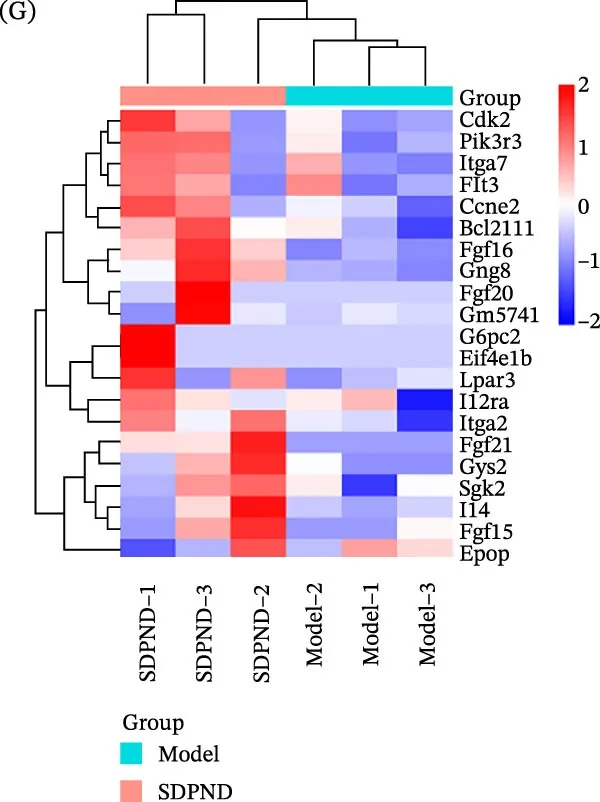

To explore the potential pathways underlying SDPND’s anti‐PD effects, GO and KEGG analyses were performed on the DEGs. GO enrichment analysis revealed significant associations with biological terms such as cytokine activity and receptor ligand activity (Figure S2). KEGG pathway analysis identified 10 significantly enriched pathways, including neuroactive ligand–receptor interaction, neurodegenerative diseases, and the MAPK signaling pathway, and DEGs were closely linked to the PI3K/AKT signaling pathway (Figure 4E). Additionally, we generated a GSEA plot and heatmap for genes related to the PI3K/AKT pathway to provide a clearer visualization of changes in these pathway genes (Figure 4F,G).

### 3.5. Prediction of SDPND Active Components and PD Targets

Potential active ingredients in SDPND were identified via UPLC‐Q‐Orbitrap‐MS analysis and integrated screening using the TCMSP and Swiss Target Prediction databases, followed by deduplication. Disease‐related genes associated with “PD” were retrieved from databases, yielding 1816 targets after duplicate removal. A total of 157 overlapping targets were collected by importing the potential active ingredient targets and PD targets of SDPND into the Venn database (Figure 5A). A “drug‐component‐target‐disease” map was constructed based on the intersecting targets (Figure 5B). These targets were imported into the STRING database, and the top seven targets were selected based on their degree of interaction (Figure 5C).

Figure 5Network construction and analysis. (A) Venn diagram of the common targets of SDPND and PD. (B) PPI protein interaction (C) core targets screening. (D) The sankey diagram of “targets‐pathways.” (E) KEGG enrichment analysis. The top 20 KEGG signaling pathways are shown. (F) KEGG enrichment analysis of top 20 genes from network pharmacology and transcriptomic intersection.

**Figure:**
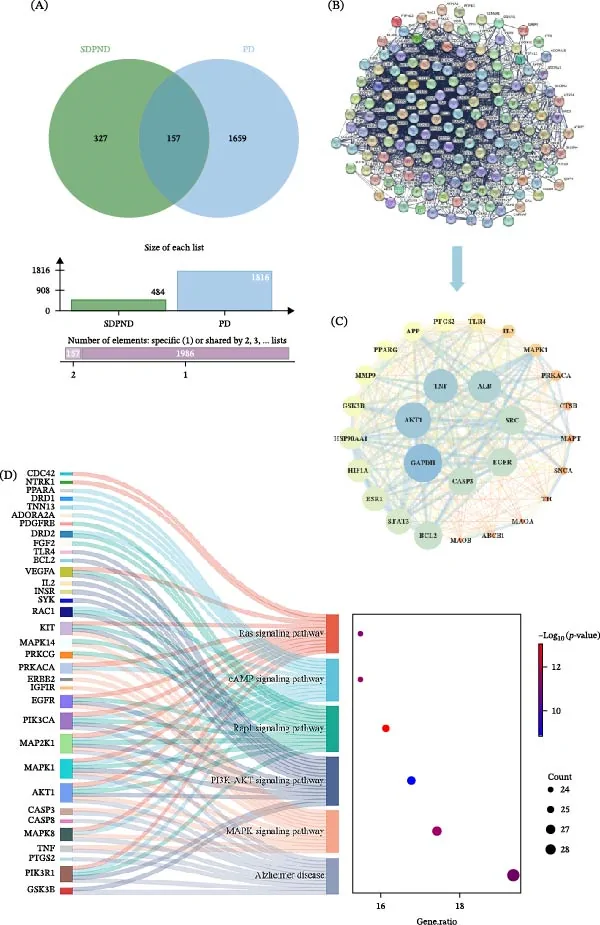

**Figure:**
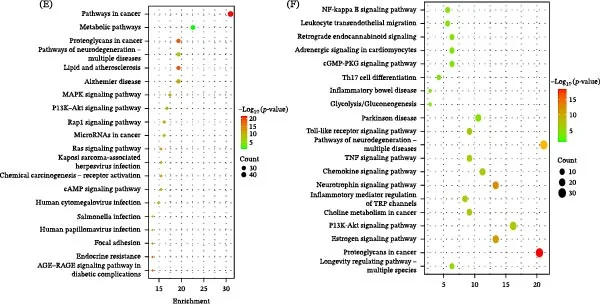

### 3.6. GO Enrichment and KEGG Pathway Analysis

GO analysis of the targets in the PPI network was performed, and the top 10 significantly enriched GO terms are shown in Figure S3. KEGG pathway analysis revealed that these targets were mainly enriched in inflammation‐ and metabolism‐related signaling pathways, including the PI3K/AKT, MAPK, and Rap1 signaling pathways (Figure 5E). Based on the KEGG results, a Sankey diagram was generated to visualize the relationships between the PI3K/AKT pathway and its associated genes (Figure 5D).

To further integrate the computational analysis with experimental findings, the intersection targets identified by network pharmacology were compared with the transcriptomic dataset. The PI3K/AKT signaling pathway was consistently enriched in both analyses (Figure 5F), suggesting that this pathway may represent a candidate mechanism for subsequent experimental validation.

### 3.7. Molecular Docking

**Figure 6:**
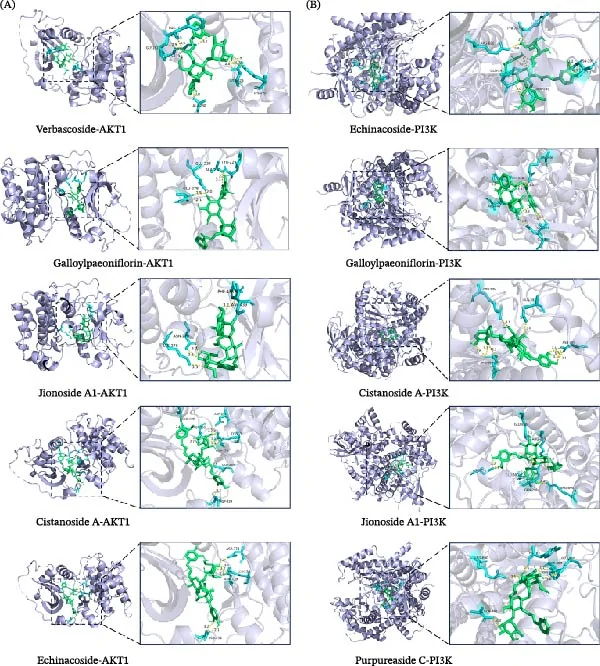
Molecular docking demonstrated binding affinities between PI3K/AKT1 and bioactive ligands. The top five compounds exhibiting the strongest binding energy for each target were visualized. (A) Visualization of the docking results between AKT1 and 5 chemical components in SDPND. (B) Visualization of the docking results between PI3K and five chemical compositions in SDPND.

Based on transcriptomic and network pharmacology analyses, molecular docking was performed to evaluate the binding potential between representative SDPND components and key proteins in the PI3K/AKT pathway. The binding affinities of the 12 selected compounds toward two core targets are summarized in Table S1, and the docking conformations of the five compounds with the strongest binding affinities are presented in Figure 6. The results demonstrated that all components exhibited binding energies ≤ −5 kcal/mol, indicating favorable binding potential. These docking results provide computational support for the possible interactions between SDPND components and PI3K/AKT‐related targets.

### 3.8. SDPND Inhibits the PI3K/AKT Pathway to Exert Anti‐Inflammatory Effects

Based on the transcriptomic, network pharmacology, and molecular docking analyses, we next performed experimental validation to determine whether PI3K/AKT signaling was functionally involved in the therapeutic effects of SDPND. To validate this finding, we conducted western blotting analysis to quantify the expression levels of PI3K, p‐PI3K, and downstream effectors, including AKT and p‐AKT, in substantia nigra and striatum tissues.

**Figure 7:**
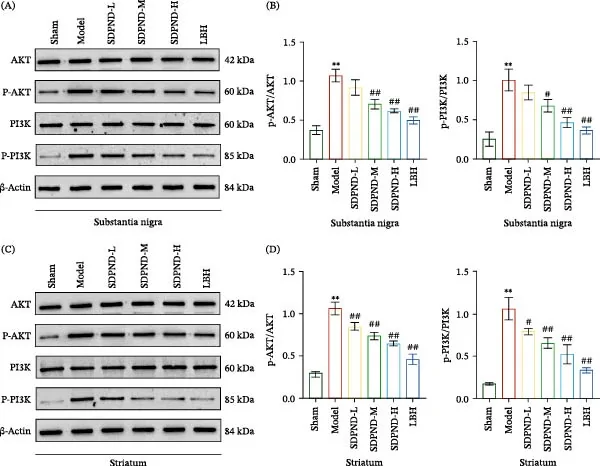
SDPND inhibits the PI3K/AKT pathway to exert anti‐inflammatory effects. Representative western blotting analysis demonstrated the protein expression of p‐PI3K, PI3K, p‐AKT, AKT, and β‐actin in substantia nigra (A) and striatum tissues (C). Relative expression ratios of p‐PI3K/PI3K and p‐AKT/AKT were quantified in the substantia nigra (B, *n* = 3) and striatum (D, *n* = 3).  ^∗^ *p* < 0.05,  ^∗∗^ *p* < 0.01, vs. Sham group; ^#^ *p* < 0.05, ^##^ *p* < 0.01, vs. Model group.

PI3K/AKT phosphorylation was significantly upregulated in substantia nigra and striatum tissues of PD model animals compared to shams (*p* < 0.01). Markedly, the observed reduction in the phosphorylation levels of PI3K and AKT clearly indicates that SDPND administration effectively suppresses the hyperactivation of this signaling pathway (Figure 7). These findings collectively demonstrated that SDPND alleviates neuroinflammatory responses by inhibiting aberrant activation of the PI3K/AKT signaling pathway.

## 4. Discussion

TCM exhibits multitarget and holistic regulatory properties, improving systemic physiological function, effectively alleviating symptoms, reducing adverse effects, and thereby enhancing the overall quality of life. These comprehensive advantages position TCM as a safer and more effective therapeutic option for PD. SDPND, a Chinese medicine formula designed to target both symptoms and root causes, demonstrates significant clinical efficacy and broad application prospects in PD treatment [27, 28]. However, elucidating its mechanisms remains challenging due to TCM’s multicomponent and multitarget nature. Therefore, understanding the chemical composition of SDPND is essential for gaining insights into its therapeutic mechanism at the molecular level. In this research, UPLC‐Q‐Orbitrap‐MS high‐resolution mass spectrometry analysis facilitated the detection of 91 well‐defined chemical constituents, with key bioactive components including catalpol, albiflorin, paeoniflorin, verbascoside, cistanoside A, galloylpaeoniflorin, and echinacoside, providing a solid foundation for further studies. Particularly, these identified compounds have been previously reported to ameliorate motor dysfunction and neuropathology in PD [11, 29–31]. For example, albiflorin has demonstrated significant therapeutic potential in PD, and experiments have revealed its ability to suppress activated microglia‐induced neuroinflammation through dual signaling pathways [32]. Paeoniflorin has been reported to ameliorate motor dysfunction in PD models and alleviate associated neuropathology. Mechanistic studies indicate that this effect may involve α‐Syn/protein kinase C δ subtype pathway regulation that reduces neuronal apoptosis [33]. Furthermore, cistanoside A has shown neuroprotective properties in PD animal models, mainly by targeting oxidative stress pathways [34]. These findings collectively demonstrate that SDPND harbors multiple anti‐PD components, substantiating its therapeutic potential.

Subsequently, pharmacological efficacy evaluations were systematically conducted, and SDPND demonstrated significant therapeutic effects in A53T mice, ameliorating motor dysfunction and attenuating neuroinflammatory infiltration in pathological assessments. Neuroinflammation, a hallmark of PD pathogenesis, is exacerbated by α‐Syn dysregulation. Under physiological conditions, α‐Syn modulates synaptic vesicle trafficking and neurotransmitter dynamics. Pathological misfolding of α‐Syn, however, generates oligomers and fibrils that aggregate intracellularly. These aggregates are released via exosomes or neuronal death, activating glial cells to secrete pro‐inflammatory cytokines that further propagate α‐Syn misfolding, neurotoxic substance accumulation, dopaminergic neurodegeneration, and PD progression [35, 36]. Microglia, the resident immune cells of the central nervous system, exhibit two distinct activation phenotypes: M1 and M2 [37, 38]. In PD pathology, microglia predominantly shift toward the M1 phenotype, contributing to neuroinflammation. Our data revealed that SDPND significantly suppressed M1 microglial activation, corroborated by ELISA‐based serum cytokine profiling. Transcriptomic analysis of A53T mice identified 652 DEGs in brain tissues, underscoring its multi‐pathway regulatory capacity. KEGG and GO enrichment analyses highlighted inflammatory pathways, including PI3K‐AKT and MAPK signaling (Figure S4). PPI network visualization further validated DEGs enrichment in immune modulation and inflammatory processes, with key nodes such as PIK3CG and TNFSF9 supporting these findings (Figure S5). Collectively, our results suggest that SDPND alleviates neuroinflammation through multipathway regulation.

Network pharmacology, as a multidisciplinary strategy synergistically integrating systems biology, computational chemistry, and polypharmacology, establishes a robust analytical framework for deciphering the bioactive constituents and mechanistic complexity of SDPND. In this investigation, we synergize network pharmacology with our previously validated pharmacological efficacy data to conduct a comprehensive investigation into the anti‐PD mechanisms. Comprehensive network analysis revealed 157 SDPND key targets for PD treatment. GO analysis emphasized their roles in signal transduction, while KEGG pathway enrichment highlighted metabolic and inflammatory pathways. Notably, cross‐validation between transcriptomics and network pharmacology confirmed PI3K/AKT pathway enrichment, reinforcing the transcriptomic findings. Molecular docking identified stable binding interactions between 12 bioactive SDPND compounds and core targets, suggesting mechanism‐driving components. Recent studies have demonstrated that several of these compounds exhibit strong interactions with the PI3K/AKT pathway, supported by robust binding affinities [39, 40]. Building on these data analyses, we will elucidate the mechanism of action of individual bioactive components in the future work. Critically, western blotting validated SDPND‐mediated downregulation of PI3K/AKT signaling and reduced phosphorylation of downstream inflammatory mediators.

The biological functions of the PI3K/AKT pathway are highly dependent on the cell type and pathological context. To further determine the cellular specificity of this regulatory effect, we performed double immunofluorescence co‐localization analyses in both brain tissues and LPS‐stimulated BV2 cells. The results demonstrated that phosphorylated PI3K and AKT were predominantly localized in Iba1‐positive microglia and that SDPND markedly reduced their activation both in vivo and in vitro. These findings provide direct evidence that the inhibitory effect of SDPND on PI3K/AKT signaling primarily occurs in activated microglia.

**Figure 8:**
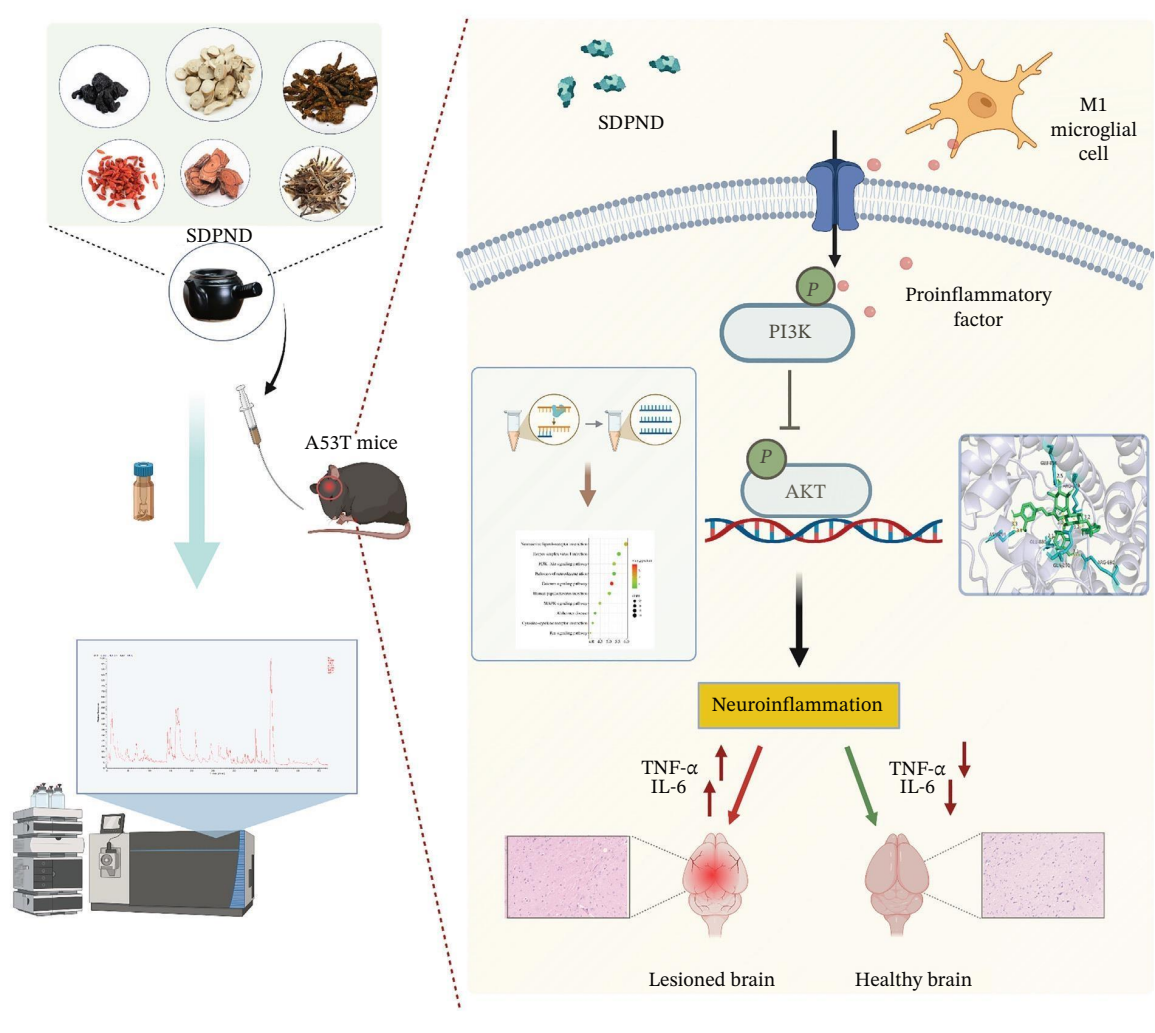
Mechanism of SDPND in the treatment of PD.

This study highlights the important role of PI3K/AKT signaling in microglia‐associated neuroinflammation during PD progression and suggests that selective regulation of this pathway in activated microglia may represent one of the mechanisms underlying the therapeutic effects of SDPND (Figure 8). Nevertheless, several limitations should be acknowledged. First, although the transcriptomic and Western blot results consistently supported our conclusions, the sample sizes used for RNA sequencing and protein validation were relatively limited, which may reduce the statistical robustness of the findings. Future studies with larger biological sample sizes are warranted to further validate these results. Second, as a multicomponent herbal formula, the pharmacological effects of SDPND likely involve coordinated regulation of multiple signaling pathways beyond PI3K/AKT signaling. Future studies should further clarify the contributions and interactions of individual bioactive constituents. In addition, more comprehensive preclinical models, including MPTP‐induced models, aged animal models, and non‐human primate models, together with systematic ADME studies focusing on blood‐brain barrier penetration and oral bioavailability, will facilitate the clinical translation of SDPND.

## 5. Conclusions

In summary, this study demonstrates that SDPND exhibits anti‐inflammatory effects and improves the motor function in A53T mice. The combined results from transcriptome sequencing, experimental validation, and network pharmacology indicate that the neuroprotective effects of SDPND are likely due to its modulation of the PI3K/AKT signaling pathway. Our work provides compelling evidence supporting SDPND as a potential alternative therapy for PD patients and lays a theoretical foundation for its clinical application.

**Nomenclature**
AKT: Protein kinase B
AKT1: Protein kinase B1
BS: Radix Paeoniae Alba
DL: *Pheretima vulgaris*
ELISA: Enzyme‐linked immunosorbent assay
GO: Gene Ontology
GQ: *Lycium barbarum*
H&E: Hematoxylin–eosin
IL‐6: Interleukin‐6
JXT: Caulis Spatholob
MAPK1: Mitogen‐activated protein kinase 1
PD: Parkinson’s disease
PI3K: Phosphatidylinositol 3‐kinase
p‐PI3K: Phosphorylation of signal transducers and activators of transcription 3
p‐AKT: Phosphorylation of protein kinase B
RCR: *Cistanche* herbal
ROS: Reactive oxygen species
SD: Radix rehmanniae preparate
SDPND: Shao Di Pa Ning Decoction
TCM: Traditional Chinese medicine
TNF‐α: Tumor necrosis factor‐alpha
α‐Syn: Alpha‐synuclein.

## Author Contributions

**Peng Huang:** methodology, writing – original draft, data curation, investigation. **Zhengqi Tao and Shuangying Gui:** validation, writing – review and editing. **Jingjing Hu:** visualization, investigation. **Wei Dong:** investigation, data curation. **Meixia Wang and Han Wang:** methodology, software. **Ting Dong:** investigation, data curation, validation. **Yun Zhang:** methodology, investigation. **Xiao Liang:** supervision, data curation. **Ning He:** supervision, methodology. **Wenming Yang and Yuzhe Huang:** project administration, funding acquisition, writing – review and editing.

## Funding

This study was supported by the national Natural Science Foundation of China (Grant 82505062), The Research Project of Traditional Chinese Medicine Inheritance and Innovation of Anhui Province (Grants 2024CCCX250 and 2024CCCX108), and The Natural Science Research Key Project of Anhui Provincial Department of Education (Grants 2024AH050988 and 2025AHGXZK30955).

## Disclosure

All data were generated in‐house, and no paper mill was used. All authors agree to be accountable for all aspects of work, ensuring integrity and accuracy.

## Ethics Statement

The Experimental Animal Ethics Committee of Anhui University of Traditional Chinese Medicine approved all experiments (Number AHUCM‐mouse‐2022026).

## Conflicts of Interest

The authors declare no conflicts of interest.

## Supporting Information

Additional supporting information can be found online in the Supporting Information section.

## Supporting information


**Supporting Information** Figure S1: Histopathological scores of the substantia nigra and striatum. Figure S2: GO enrichment analysis of DEGs in transcriptome. Figure S3: GO enrichment in network pharmacology. Figure S4: Metabolic pathway enrichment associated with PI3K/AKT. Figure S5: PPI network of DEGs in the transcriptome. Table S1: Binding affinities and predicted inhibition constants (pKi) of molecular docking.

## Data Availability

The data that support the findings of this study are available from the corresponding author upon reasonable request.
