# Supplementary material for: Shao Di Pa Ning Decoction Attenuates Neuroinflammation in Parkinson’s Disease via PI3K/AKT Signaling Pathway
Source: Mediators Inflamm. 2026 Aug 30;2026:6450498. doi: 10.1155/mi/6450498 (PMC13526254; doi:10.1155/mi/6450498)
Supplement: Supplementary file 1 [file MI-2026-6450498-s001.docx]

Fig. S1: Histopathological Scores of the Substantia Nigra and Striatum;

Fig. S2. GO enrichment analysis of DEGs in transcriptome.

Fig. S3. GO enrichment in network pharmacology.

Fig. S4. Metabolic pathway enrichment associated with PI3K/AKT.

Fig. S5. PPI network of DEGs in the transcriptome.

Table. S1. Binding affinities and predicted inhibition constants (pKi) of molecular docking

| Protein | AKT | | PI3K | |
| --- | --- | --- | --- | --- |
| Compounds | Binding affinity (kcal/mol) | pKi (μM) | Binding affinity (kcal/mol) | pKi (μM) |
| 5-Ethyl-2-pentylfuran-3-sulfonic.acid | -6.03±0.12 | -4.43±0.08 | -6.60±0.10 | 4.84±0.07 |
| Albiflorin | -8.27±0.25 | 6.60±0.18 | -8.70±0.17 | 6.38±0.13 |
| Catalpol | -7.30±0.10 | 5.35±0.07 | -7.57±0.23 | 5.55±0.17 |
| Cistanoside.A | -9.83±0.21 | 7.21±0.158 | -10.53±0.15 | 7.73±0.11 |
| Echinacoside | -9.77±0.21 | 7.16±0.15 | -10.73±0.55 | 7.87±0.40 |
| galloylpaeoniflorin | -10.60±0.44 | 7.77±0.32 | -10.60±0.26 | 7.77±0.19 |
| Jionoside.A1 | -10.03±0.49 | 7.36±0.36 | -10.50±0.70 | 7.70±0.51 |
| Paeoniflorin | -8.47±0.15 | 6.21±0.11 | -9.00±0.36 | 6.60±0.26 |
| Purpureaside.C | -9.37±0.21 | 6.87±0.15 | -10.23±0.71 | 7.51±0.52 |
| Rehmannioside.C | -7.87±0.12 | 5.77±0.08 | -8.43±0.32 | 6.19±0.24 |
| Tubuloside.A | -9.00±0.30 | 6.60±0.22 | -10.13±0.67 | 7.43±0.49 |
| Verbascoside | -10.80±0.17 | 7.92±0.13 | -9.37±0.06 | 6.87±0.04 |
